# Exploring the clinical value of preoperative serum gamma-glutamyl transferase levels in the management of patients with hepatocellular carcinoma receiving postoperative adjuvant transarterial chemoembolization

**DOI:** 10.1186/s12885-021-08843-z

**Published:** 2021-10-18

**Authors:** Qiao Ke, Fu Xiang, Chunhong Xiao, Qizhen Huang, Xiaolong Liu, Yongyi Zeng, Lei Wang, Jingfeng Liu

**Affiliations:** 1 Department of Hepatopancreatobiliary Surgery, Mengchao Hepatobiliary Hospital of Fujian Medical University, Fuzhou, Fujian China; 2 Department of General Surgery, the First Affiliated Hospital of Dalian Medical University, Dalian, Liaoning China; 3 Department of General Surgery, 900th Hospital of PLA, Fuzhou, Fujian China; 4 Department of Radiation Oncology, Mengchao Hepatobiliary Hospital of Fujian Medical University, Fuzhou, Fujian China; 5 The United Innovation of Mengchao Hepatobiliary Technology Key of Fujian Province, Mengchao Hepatobiliary Hospital of Fujian Medical University, Fuzhou, Fujian China; 6 Department of Radiation Oncology, Fujian Cancer Hospital, Fujian Medical University Cancer Hospital, Fuzhou, Fujian China

**Keywords:** Hepatocellular carcinoma, Gamma-glutamyl transferase, Radical resection, Transarterial chemoembolization

## Abstract

**Background:**

Preoperative serum gamma-glutamyl transferase (γ-GT) levels is significantly related to the prognosis of hepatocellular carcinoma (HCC), but its clinical value in the management of postoperative adjuvant transarterial chemoembolization (PA-TACE) has rarely been explored. This study aimed to investigate whether γ-GT levels could be taken as a biomarker to guide the management of PA-TACE in resectable HCC.

**Methods:**

HCC patients receiving radical resection were identified through the primary liver cancer big data (PLCBD) from December 2012 to December 2015. Prognostic factors of overall survival (OS) and disease-free survival (DFS) were identified by univariate and multivariate cox analyses, and subgroup analysis was conducted between PA-TACE group and non-TACE stratified by γ-GT levels before and after 1:1 propensity score matching (PSM).

**Results:**

γ-GT level was found to be an independent risk factor of OS and DFS in 1847 HCC patients receiving radical resection (both *P <* 0.05), and patients with elevated γ-GT(> 54.0 U/L) have a shortened median OS and DFS, compared with those with normal γ-GT (both *P* < 0.001). In the subgroup of patients with normal γ-GT, there were no significant differences between groups of PA-TACE and non-TACE in terms of median OS and DFS before and after PSM (all *P >* 0.05), and PA-TACE was not a significant prognostic factor of both OS and DFS before and after PSM (all *P >* 0.05). In the subgroup of patients with elevated γ-GT, significant differences were found between groups of PA-TACE and non-TACE in terms of median OS and DFS before and after PSM (all *P <* 0.05), and PA-TACE was an independent prognostic factor of both OS and DFS (all *P <* 0.05).

**Conclusion:**

Currently, we concluded that patients with more advanced HCC also have more elevated γ-GT, and these patients with elevated γ-GT would be benefited more from PA-TACE after radical resection.

**Supplementary Information:**

The online version contains supplementary material available at 10.1186/s12885-021-08843-z.

## Background

Hepatocellular carcinoma (HCC) is still one of the most common malignancies globally [1, 2], and approximately 854,000 patients have been newly diagnosed as HCC per year [3]. But the prognosis remains unfavorable with the median overall survival of 30 to 40 months [3], regardless of substantial advances in the early detection, treatment and surveillance. Radical resection has been still the most cost-effective curative treatment for patients with HCC [4, 5], but the long-term prognosis remains far from satisfactory with the 5-year recurrence rate up to 70% [1]. Hence, strategies designed to prevent the recurrence are badly warranted in clinic.

Transarterial chemoembolization (TACE) is typically considered to be the first-line treatment for unresectable HCC according to the current guidelines [3, 4], but it has been also tried prevalently to prevent the recurrence of patients receiving resection, especially in China [6–8]. A number of studies found that postoperative adjuvant TACE (PA-TACE) could decrease the incidence of early recurrence and improve the long-term prognosis [9, 10], but worries on its efficacy have never lessen: 1) PA-TACE was found not benefit for all patients receiving resection [11], 2) adverse events (AE) related to TACE was unavoidable [12], and 3) PA-TACE might potentially cause distant metastasis [13, 14]. Hence, identifying the potential beneficiaries from PA-TACE is the key.

Gamma-glutamyl transferase (γ-GT) is a cell-membrane-bound enzyme modulating the metabolic process of glutathione (GSH) [15]. which is well concerned in the prognosis of tumors mainly because it is non-invasive and easily acquired. In the recent two systematic review and meta-analysis [16, 17], preoperative γ-GT levels is confirmed to be considerably correlated with the unfavorable clinicopathological characteristics and poor prognosis of HCC patients. To the best of our knowledge, serum GGT has been reported open in the management of palliative TACE for advanced HCC [18] and PA-TACE for resectable intrahepatic cholangiocarcinoma [19], but there are seldom reports about serum γ-GT guiding the management of PA-TACE for resectable HCC. Therefore, we extracted the data from the primary liver cancer big data (PLCBD), which was designed to collect data on primary liver cancer from multi-centers in China, to identify it.

## Methods

### Patient selection

This study was approved by Mengchao Hepatobiliary Hospital of Fujian Medical University’s Ethics Committee (No. 2019_039_01) under the guideline of the 1975 Declaration of Helsinki. Informed consent was signed by all patients before any clinical intervention. Data of HCC patients receiving radical resection between December 2012 and December 2015 including age, sex, preoperative serum levels of alpha-fetoprotein (AFP), total bilirubin (TBil) and γ-GT level, tumor features confirmed by pathology, and follow-up was extracted from PLCBD by an IT engineer, and then was checked by three independent researchers.

Patients were enrolled into this study if they underwent a radical resection and were diagnosed as HCC by postoperative pathology, and the radical resection criterion was the same as previously depicted [20]. Patients who received hepatectomy for recurrent HCC, preoperative treatments, or had macrovascular invasion, bile duct invasion, or died within one month following hepatectomy were excluded from this current study.

### Interventions

PA-TACE was generally carried out 6.0 (4.0–8.0) weeks after radical hepatectomy. Briefly, a 5-F catheter was inserted into the selective hepatic artery under the guide of the digital shadow angiography (DSA), and then chemotherapeutics agents were slowly injected followed by an emulsion of iodized oil (2-5 ml). The preferred regimen was cisplatin (10–30 mg), doxorubicin hydrochloride (10 mg) or pharmorubicin (20–40 mg), but the dosages were calculated by the remaining liver volume and body surface [21, 22].

### Follow-up

All patients underwent a comprehensive evaluation of blood routine analysis, biochemical index, AFP levels, and contrast-enhanced computed tomography (CT) or magnetic resonance imaging (MRI) at one month after surgery. Then, patients received routine blood test, physical examination, and abdominal ultrasonography every three months in the first 2 year after surgery, every six months from 2 to 5 years, and every 12 months after 5 years according to the guideline [5]. Any suspected recurrence or metastasis should be confirmed by contrast enhanced CT or MRI, and once confirmed, further treatment such as repeat hepatectomy, TACE, radiofrequency ablation, should be immediately adopted.

### Endpoints

The endpoints were overall survival (OS) and disease-free survival (DFS). OS time was determined from the data of resection to either the data of death or the latest follow-up. DFS time was calculated from the data of resection to the date of recurrence or the date of the latest follow-up.

### Statistics

Clinicopathological variables were selected according to the previous reports [20, 23]. Specially, the value of serum levels of γ-GT(≤54.0 or > 54.0 U/L) were categorized using the upper limit of the normal values in our hospital. Tumor differentiation was determined by the Edmondson-Steiner grading system according to the highest grade in a specimen [21].

The survival curves of OS and DFS were determined by the Kaplan-Meier method in a whole cohort, and independent risk factors were identified by the forward method of the multivariate Cox regression model.

The whole cohort was then divided into two subgroups according to the levels of γ-GT (≤54.0 or > 54.0 U/L). The efficacy of PA-TACE was evaluated in each subgroup using Kaplan-Meier method before and after a well-designed 1:1 propensity score matching (PSM), and the adjusted factors were age, TBil, AFP, tumor number, tumor diameter, Edmondson-Steiner grading, capsule, satellite, and MVI, which was performed as previously reported [24]. Finally, independent risk factors associated with OS and DFS were examined by a multivariate Cox regression model in each subgroup before and after PSM.

The statistical analysis was conducted using Rstudio including packages of “Table 1”, “MatchIt”, “survminer”, and “survival”. All statistical tests were two-sided, and *P* < 0.05 was considered statistically significant in this study.

**Table 1:** Clinical and pathological characteristics of the whole cohort

Characteristics		Value
**Age** (years)	Mean (SD)	52.2 (10.6)
**Sex**	Male	1575 (85.3%)
	Female	272 (14.7%)
**HBV infection**	No	242 (13.1%)
	Yes	1605 (86.9%)
**Cirrhosis**	No	610 (33.0%)
	Yes (compensated/decompensated)	1216 (65.9%)/21 (1.1%)
**TBil (**μmol/L**)**	Mean (SD)	14.6 (6.2)
**Child-pugh**	A	1751 (94.8%)
	B	96 (5.2%)
**AFP**	≤400 ng/mL	1255 (67.9%)
	> 400 ng/mL	592 (32.1%)
**γ-GT**	≤54 U/L	947 (51.3%)
	> 54 U/L	900 (48.7%)
**Transfusion**	No	1739 (94.2%)
	Yes	108 (5.8%)
**Tumor number**	Single	1499 (81.2%)
	Multiple	348 (18.8%)
**Tumor diameter** (cm)	Mean (SD)	5.8 (3.8)
**ES grading**	I&II	172 (9.3%)
	III&IV	1675 (90.7%)
**Capsule**	Present	1487 (80.5%)
	Absent	360 (19.5%)
**Satellite**	No	1071 (58.0%)
	Yes	776 (42.0%)
**MVI**	Present	644 (34.9%)
	Absent	1203 (65.1%)
**BCLC**	0	107 (5.8%)
	A	1446 (78.3%)
	B	294 (15.9%)
**AJCC**	Ia	107 (5.8%)
	Ib	938 (50.8%)
	II	623 (33.7%)
	IIIa	179 (9.7%)
**CNLC**	Ia	846 (45.8%)
	Ib	707 (38.3%)
	IIa	203 (11.0%)
	IIb	91 (4.9%)
**PA-TACE**	No	1335 (72.3%)
	Yes	512 (27.7%)

Note: *HBV* hepatitis B virus, *TBil* total bilirubin; *AFP* alpha-fetoprotein; *γ-GT* gamma-glutamyl transferase; *ES* Edmondson-Steiner; *MVI* microvascular invasion; *PA-TACE* postoperative adjuvant transarterial chemoembolization; *AJCC* according to the 8th American joint committee on cancer staging; *CN* CN staging was defined according the Chinese guideline for HCC

## Results

### Clinicopathological characteristics of patients

**Fig. 1:**
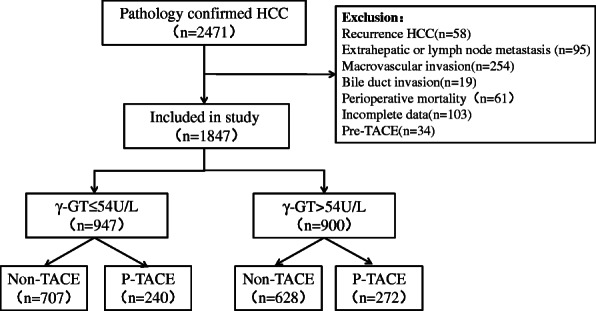
Flow chart of patients’ enrollment from the primary liver cancer big data

Initially, 2471 HCC patients were confirmed by pathology. After excluding 642 patients according to the exclusion criteria, 1847 patients remained to be further analyzed, including 974 patients (51.3%) with γ-GT ≤ 54 U/L and 900 patients (48.7%) with γ-GT > 54 U/L, respectively (Fig. 1).

The clinicopathological characteristics of all the 1847 patients were listed in Table 1. 1237 patients (77.0%) were present with cirrhosis, which might because 86.9% patients were found to be infected by HBV previously or currently. The average tumor diameter was 5.8 ± 3.8 cm, 348 patients (18.8%) were found to be with multiple tumors, and 644 patients (34.9%) with MVI.

### Prognostics factors of HCC patients after radical resection

The median follow-up was 36 months. In whole cohort, the median OS was 59.7 months, and the 1-, 3-, 5-year survival rates were 91.2, 71.3, 63.4%, respectively. The median DFS was 38.4 months, and the 1-, 3-, 5-year DFS rates were 76.5, 53.1, 46.7%, respectively. Kaplan Meier survival analysis showed that no significant differences were observed between PA-TACE group and non-TACE group in terms of median OS (60.0 months vs. 59.0 months, *P* = 0.160, Supplement Fig. 1A) and DFS (40.0 months vs. 38.0 months, *P* = 0.280, Supplement Fig. 1B). Multivariate analysis showed that AFP > 400 ng/mL, γ-GT > 54 U/L, multiple tumors, tumor diameter and presence of MVI were identified to be independent risk factors of OS and DFS (all *P* < 0.05, Table 2). Of note, PA-TACE was not found to be associated with OS and DFS (both *P* > 0.05, Table 2).

**Table 2:** Univariate and multivariate analysis of overall survival and disease-free survival in the whole cohort

Characteristics	OS				DFS			
	Univariate		Multivariate		Univariate		Multivariate	
	HR (95CI)	***P-***value	HR (95CI)	***P-***value	HR (95CI)	***P-***value	HR (95CI)	***P-***value
**Age** (years)	1.00 (0.99–1.01)	0.818			1.00 (0.99–1.01)	0.852		
**Sex** (Male vs Female)	0.95 (0.77–1.16)	0.611			0.99 (0.84–1.18)	0.950		
**HBV infection (**Yes vs No)	1.01 (0.80–1.28)	0.908			1.13 (0.93–1.38)	0.208		
**Cirrhosis (**Yes vs No)	0.95 (0.81–1.11)	0.498			1.08 (0.95–1.24)	0.237		
**TBil** (μmol/L)	1.02 (1.01–1.03)	0.003	1.01 (1.00–1.02)	0.087	1.01 (1.00–1.02)	0.010	1.01 (1.00–1.02)	0.186
**Child-pugh** (B vs A)	2.04 (1.54–2.70)	< 0.001	1.27 (0.93–1.72)	0.131	1.72 (1.34–2.20)	< 0.001	1.13 (0.86–1.49)	0.371
**AFP** (> 400 vs 400 ng/mL)	1.66 (1.43–1.94)	< 0.001	1.43 (1.22–1.67)	< 0.001	1.35 (1.19–1.54)	< 0.001	1.22 (1.07–1.40)	0.004
**γ-GT** (> 54 vs ≤54 U/L)	1.76 (1.51–2.05)	< 0.001	1.38 (1.17–1.62)	< 0.001	1.69 (1.49–1.92)	< 0.001	1.42 (1.24–1.63)	< 0.001
**Transfusion (**Yes vs No)	2.01 (1.54–2.61)	< 0.001	0.89 (0.66–1.22)	0.479	1.97 (1.57–2.48)	< 0.001	1.12 (0.86–1.46)	0.391
**Tumor number (**Multiple vs Single)	1.83 (1.54–2.17)	< 0.001	1.50 (1.23–1.82)	< 0.001	1.82 (1.58–2.10)	< 0.001	1.53 (1.29–1.80)	< 0.001
**Tumor diameter (**cm)	1.11 (1.09–1.13)	< 0.001	1.07 (1.05–1.09)	< 0.001	1.08 (1.06–1.09)	< 0.001	1.04 (1.02–1.06)	< 0.001
**ES grading (**III/IV vs I/II)	1.80 (1.34–2.42)	< 0.001	1.28 (0.95–1.74)	0.108	1.42 (1.13–1.78)	0.003	1.08 (0.85–1.37)	0.528
**Capsule (**Absent vs Present)	1.42 (1.20–1.70)	< 0.001	1.35 (1.12–1.62)	0.001	1.26 (1.09–1.47)	0.002	1.16 (0.99–1.35)	0.064
**Satellite (**Yes vs No)	1.51 (1.30–1.75)	< 0.001	0.98 (0.81–1.18)	0.812	1.46 (1.29–1.66)	< 0.001	1.05 (0.90–1.22)	0.527
**MVI (**Absent vs Present)	1.92 (1.65–2.23)	< 0.001	1.51 (1.27–1.79)	< 0.001	1.68 (1.48–1.90)	< 0.001	1.40 (1.22–1.62)	< 0.001
**PA-TACE (**Yes vs No)	0.89 (0.75–1.05)	0.165			0.93 (0.81–1.06)	0.280		

Note: *OS* overall survival; *DFS* disease-free survival; *HR* hazard ratio; *CI* confidence interval; *HBV* hepatitis B virus, *TBil* total bilirubin; *AFP* alpha-fetoprotein; *γ-GT* gamma-glutamyl transferase; *ES* Edmondson-Steiner; *MVI* microvascular invasion; *PA-TACE* postoperative adjuvant transarterial chemoembolization

### Relationship between patients’ γ-GT level and clinical characteristics

All patients were divided into two subgroups according to the γ-GT levels. 947 patients (51.3%) were identified as normal γ-GT group with γ-GT ≤ 54 U/L, and 900 patients (48.7%) were elevated γ-GT group with γ-GT > 54 U/L. The proportions of male, HBV infection, TBil level, Child-pugh class B, intraoperative transfusion, tumor diameter, the percentages of multiple tumors, and advanced stages (BCLC stage B, AJCC stage IIIa, and CNLC stage IIb) were apparently higher in the elevated γ-GT group than those in the normal γ-GT group (all *P* < 0.05, Table 3). Importantly, patients with elevated γ-GT were much more likely to receive PA-TACE than those with normal γ-GT (*P* < 0.05, Table 3). As expected, the pooled HR for the median OS was in favor of patients with normal γ-GT, compared with those with elevated γ-GT (65.9 months vs. 55.8 months, *P* < 0.001, Supplement Fig. 2A); similar difference was observed in median DFS (53.8 months vs. 25.3 months, *P* < 0.001, Supplement Fig. 2B).

**Table 3:** Clinicopathological characteristics according to the level of **γ**-GT

Characteristic		γ-GT ≤ 54 U/L (***n*** = 947)	γ-GT > 54 U/L (***n*** = 900)	***P***-value
Age (years)	Mean ± SD	52.3 (10.5)	52.1 (10.6)	0.778
**Sex**	Female	182 (19.2%)	90 (10.0%)	< 0.001
	Male	765 (80.8%)	810 (90.0%)	
**HBV infection**	No	139 (14.7%)	103 (11.4%)	0.047
	Yes	808 (85.3%)	797 (88.6%)	
**Cirrhosis**	No	315 (33.3%)	295 (32.8%)	0.863
	Yes	632 (66.7%)	605 (67.2%)	
**TBil** (μmol/L)	Mean ± SD	14.2 (5.54)	15.1 (6.78)	0.003
**Child-pugh**	A	925 (97.7%)	826 (91.8%)	< 0.001
	B	22 (2.3%)	74 (8.2%)	
**AFP** (ng/mL)	≤400	662 (69.9%)	593 (65.9%)	0.072
	> 400	285 (30.1%)	307 (34.1%)	
**Transfusion**	No	926 (97.8%)	813 (90.3%)	< 0.001
	Yes	21 (2.2%)	87 (9.7%)	
**Tumor number**	Single	799 (84.4%)	700 (77.8%)	< 0.001
	Multiple	148 (15.6%)	200 (22.2%)	
**Tumor diameter** (cm)	Mean ± SD	4.60 (2.64)	7.17 (4.33)	< 0.001
**ES grading**	I/II	99 (10.5%)	73 (8.1%)	0.097
	III/IV	848 (89.5%)	827 (91.9%)	
**Capsule**	Present	770 (81.3%)	717 (79.7%)	0.405
	Absent	177 (18.7%)	183 (20.3%)	
**Satellite**	No	572 (60.4%)	499 (55.4%)	0.035
	Yes	375 (39.6%)	401 (44.6%)	
**MVI**	Present	311 (32.8%)	333 (37.0%)	0.068
	Absent	636 (67.2%)	567 (63.0%)	
**BCLC**	0	79 (8.3%)	28 (3.1%)	< 0.001
	A	755 (79.8%)	691 (76.8%)	
	B	113 (11.9%)	181 (20.1%)	
**AJCC**	Ia	79 (8.3%)	28 (3.1%)	< 0.001
	Ib	495 (52.3%)	443 (49.2%)	
	II	322 (34.0%)	301 (33.4%)	
	IIIa	51 (5.4%)	128 (14.2%)	
**CNLC**	Ia	549 (58.0%)	297 (33.0%)	< 0.001
	Ib	285 (30.1%)	422 (46.9%)	
	IIa	90 (9.5%)	113 (12.6%)	
	IIb	23 (2.4%)	68 (7.6%)	
**PA-TACE**	No	707 (74.7%)	628 (69.8%)	0.022
	Yes	240 (25.3%)	272 (30.2%)	

Note: *HBV* hepatitis B virus, *TBil* total bilirubin; *AFP* alpha-fetoprotein; *γ-GT* gamma-glutamyl transferase; *ES* Edmondson-Steiner; *MVI* microvascular invasion; *PA-TACE* postoperative adjuvant transarterial chemoembolization; *AJCC* according to the 8th American joint committee on cancer staging; *CNLC* CNLC staging was defined according the Chinese guideline for HCC

### The relationship between γ-GT level and the prognosis of patients

**Fig. 2:**
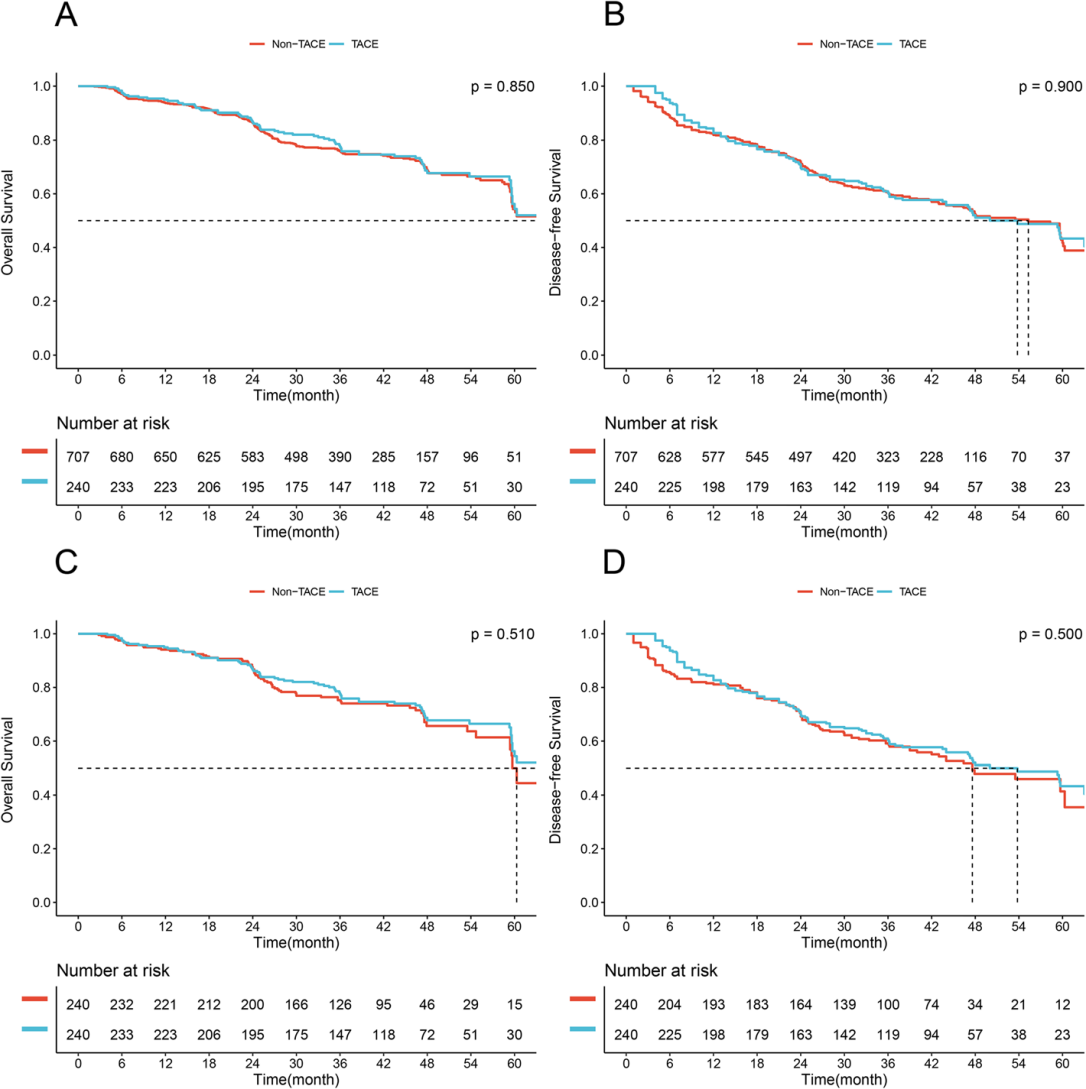
Comparison of overall survival and disease-free survival between the PA-TACE and non-TACE groups in HCC patients with γ-GT ≤ 54 U/L. (**A**, **B**), Overall survival and disease-free survival before PSM. (**C**, **D**), Overall survival and disease-free survival after PSM

In the normal γ-GT group, 240 patients (25.3%) received PA-TACE and 707 received surgery alone (Fig. 1). Significant differences were not observed between groups of PA-TACE and non-TACE in terms of median OS and DFS (65.9 months vs. 64.8 months, *P* = 0.850, Fig. 2A; 53.8 months vs. 55.3 months, *P* = 0.900, Fig. 2B; respectively). Similar result was observed in the median OS and DFS between groups of PA-TACE and non-TACE after 1:1 PSM (65.9 months vs. 60.3 months, *P* = 0.510, Fig. 2C, 53.8 months vs. 47.6 months, *P* = 0.500, Fig. 2D; respectively). Clinicopathological characteristics of patients with γ-GT ≤ 54 U/L receiving PA-TACE or not before and after PSM were depicted in supplement Table 1, and the baselines were well-balanced in two groups after PSM. Multivariate cox regression analyses showed that PA-TACE was not an independent risk factor of both OS and DFS before and after PSM (all *P* > 0.05, Table 4).

**Table 4:** Multivariate Cox regression analyses of the overall survival and disease-free survival according to the level of γ-GT before and after PSM

Characteristics	OS						DFS	
	Before PSM		After PSM		Before PSM		After PSM	
	HR (95CI)	***P-***value	HR (95CI)	***P-***value	HR (95CI)	***P-***value	HR (95CI)	***P-***value
**γ-GT ≤ 54 U/L**								
**Tumor number** (Multiple vs Single)	2.11 (1.55–2.87)	< 0.001	1.97 (1.33–2.93)	0.001	2.07 (1.61–2.65)	< 0.001	2.18 (1.59–2.98)	< 0.001
**Tumor diameter** (cm)	1.11 (1.07–1.15)	< 0.001	1.11 (1.05–1.17)	< 0.001	1.08 (1.04–1.12)	< 0.001	1.08 (1.03–1.13)	0.003
**Capsule** (Absent vs Present)	1.36 (1.02–1.81)	0.039	1.41 (0.95–2.09)	0.085	–	–	–	–
**MVI** (Absent vs Present)	1.58 (1.22–2.06)	0.001	1.57 (1.10–2.26)	0.014	1.44 (1.16–1.79)	0.001	1.45 (1.08–1.95)	0.014
**PA-TACE** (Yes vs No)	0.85 (0.65–1.11)	0.222	0.89 (0.64–1.23)	0.471	0.86 (0.69–1.08)	0.190	0.91 (0.71–1.18)	0.483
**γ-GT > 54 U/L**								
**TBil** (μmol/L)	1.01 (1.00–1.03)	0.036	1.02 (1.01–1.04)	0.009	1.01 (1.00–1.03)	0.028	1.03 (1.01–1.05)	< 0.001
**AFP** (> 400 vs 400 ng/mL)	1.71 (1.39–2.10)	< 0.001	1.71 (1.32–2.22)	< 0.001	1.43 (1.20–1.71)	< 0.001	1.47 (1.18–1.84)	0.001
**Tumor number** (Multiple vs Single)	–	–	–	–	1.25 (1.01–1.56)	0.042	1.08 (0.82–1.42)	0.589
**Tumor diameter** (cm)	1.06 (1.04–1.09)	< 0.001	1.07 (1.04–1.10)	< 0.001	1.03 (1.01–1.05)	0.008	1.04 (1.01–1.06)	0.005
**Capsule** (Absent vs Present)	1.33 (1.05–1.68)	0.019	1.43 (1.05–1.95)	0.024	–	–	–	–
**MVI** (Absent vs Present)	1.43 (1.14–1.79)	0.002	1.21 (0.89–1.64)	0.219	1.37 (1.13–1.65)	0.001	1.19 (0.92–1.54)	0.174
**PA-TACE** (Yes vs No)	0.69 (0.55–0.86)	0.001	0.66 (0.52–0.85)	0.001	0.76 (0.63–0.91)	0.003	0.74 (0.60–0.92)	0.006

Note: *OS* overall survival; *DFS* disease-free survival; *HR* hazard ratio; *CI* confidence interval; *PSM* propensity score matching; *γ-GT* gamma-glutamyl transferase; *TBil* total bilirubin; *AFP* alpha-fetoprotein; *MVI* microvascular invasion; *PA-TACE* postoperative adjuvant transarterial chemoembolization

**Fig. 3:**
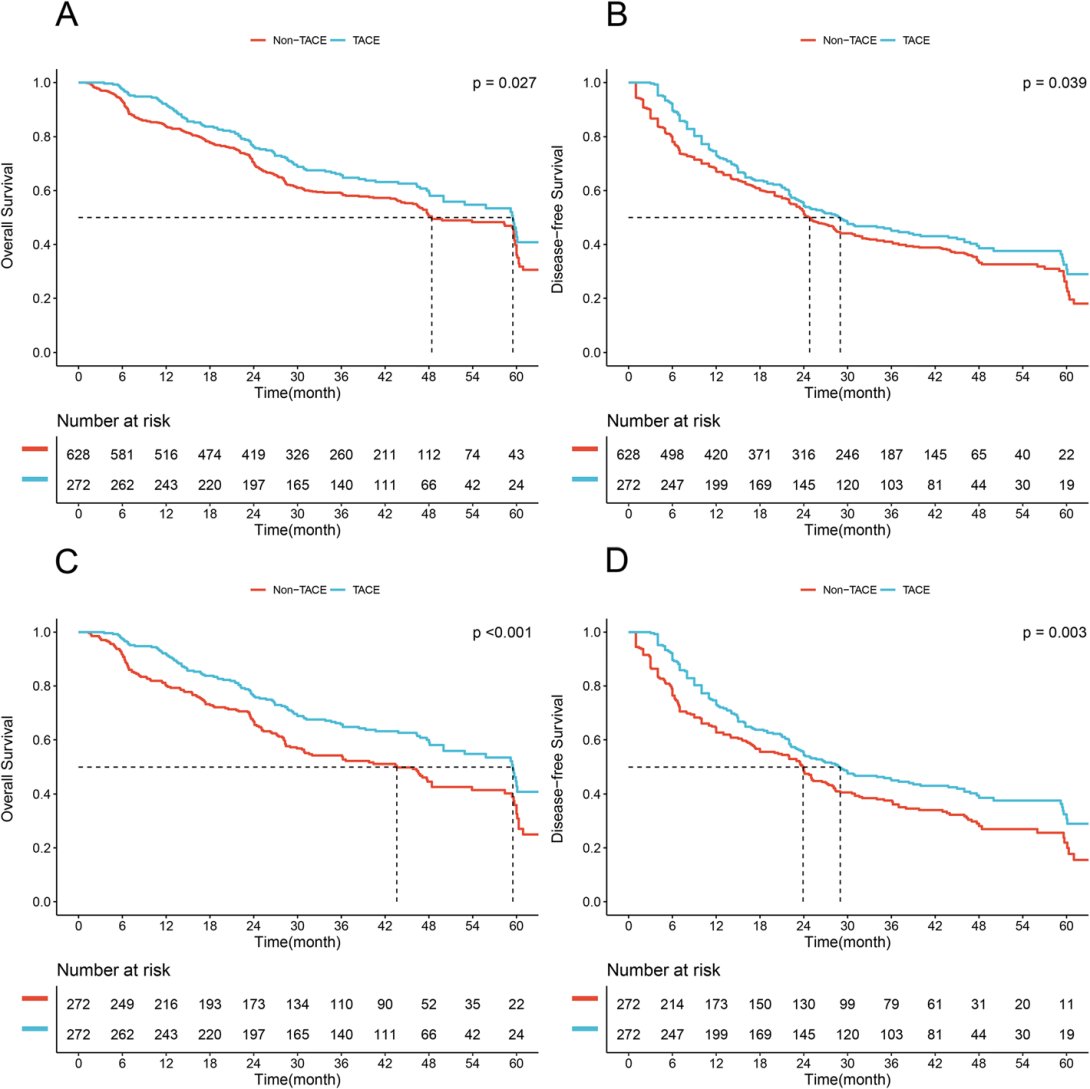
Comparison of overall survival and disease-free survival between the PA-TACE and non-TACE groups in HCC patients with γ-GT > 54 U/L. (**A**, **B**), Overall survival and disease-free survival before PSM. (**C**, **D**), Overall survival and disease-free survival after PSM

In the elevated γ-GT group, 272 patients (30.2%) received PA-TACE and 628 received surgery alone (Fig. 1). Median OS and DFS were significantly longer in the subgroup of PA-TACE than those in the subgroup of non-TACE (59.5 months vs. 48.4 months, *P* = 0.027, Fig. 3A; 29.0 months vs. 24.8 months, *P* = 0.039, Fig. 3B; respectively), which were confirmed after 1:1 PSM (59.5 months vs. 43.6 months, *P* < 0.001, Fig. 3C; 29.0 months vs. 23.9 months, *P* = 0.003, Fig. 3D; respectively). Clinicopathological characteristics of patients with γ-GT > 54 U/L receiving PA-TACE or not before and after PSM were depicted in supplement Table 2, and the baselines were well-balanced in two groups after PSM. Of note, PA-TACE was found to be an independent prognostic factor of both OS and DFS before and after PSM (all *P* < 0.05, Table 4).

## Discussion

γ-GT is an emerging biomarker for HCC early detection and prognosis prediction [25, 26], but its clinical value is far from being applied. In the current study, we found that elevated preoperative γ-GT was associated with worse OS and DFS, and was also correlated with aggressive tumor characteristics and increasing risk of intraoperative transfusion. In addition, PA-TACE could prolong the median OS and DFS of patients with elevated γ-GT before and after PSM, which was also an independent risk factor of both OS and DFS. Hence, γ-GT could be taken as an alternative biomarker to guide the management of PA-TACE.

PA-TACE is often conducted to eradicate the microscopic tumor sites [20, 27], which are either independent from primary tumor size or are not removed completely by hepatectomy, but it remains controversial whether PA-TACE could benefit patients with HCC after radical resection. In the current study, no significant differences were observed in terms of OS and DFS between groups of receiving PA-TACE or not (both *P* > 0.05), which was similar with previous reports [27–29]. Reasons are mainly because most of the published results are retrospective and patients receiving PA-TACE are typically present with more aggressive tumor characteristics and worse performance status [29, 30]. Hence, PA-TACE should be recommended with cautious to patients with “high risk factors”, but the key is to identify those who would be benefited from PA-TACE.

γ-GT is reported to improve tumor development and progression [31, 32], which could be regarded as an alternative biomarker of HCC diagnosis, especially for those with clinically negative AFP [33]. γ-GT is also found to be correlated with clinicopathological characteristics and prognosis of HCC. In the current study, elevated γ-GT was found to be associated with the incidence of rising TBil, HBV infection, intraoperative transfusion, multiple tumors, tumor diameter, satellite, and advanced stages (BCLC staging B, AJCC staging IIIa, and CNLC staging II), which indicated that it could be considered to be an noninvasive predictor of prognosis [34, 35]. In addition, γ-GT levels was found to be an independent risk factor of both OS and DFS as well as AFP (all *P* < 0.05), which indicated that γ-GT levels could also be taken as postoperative monitoring index. Hence, γ-GT might be taken as a promising biomarker to guide the performance of PA-TACE.

In fact, γ-GT has been identified to be associated with prognosis of patients receiving TACE as an initial treatment, and baseline γ-GT levels has also been found to be a significant prognostic factor for patients with intermediate HCC receiving TACE and conformal radiotherapy [15, 36]. But the clinical value of γ-GT levels to guide the performance of PA-TACE has rarely been explored. In the current study, patients with elevated γ-GT were much more likely to receive PA-TACE than those with normal γ-GT (*P* < 0.05), and only patients with elevated γ-GT but not normal γ-GT were found to be benefited from PA-TACE before and after PSM. In addition, PA-TACE was identified as independent protective factor of both OS and DFS (both *P* < 0.05), which indicated that our conclusion was robust. Hence, γ-GT could be taken as an alternative biomarker to guide the performance of PA-TACE, and patents with elevated γ-GT should be recommended to receive PA-TACE.

The mechanism of γ-GT levels to predict the efficacy of TACE, in our opinion, lies on its interaction with liver microenvironment. As a membrane-bound enzyme, γ-GT is an essential element for the production of intracellular glutathione (GSH), which could prevent the tumor cell from damage of reactive oxygen species (ROS) and free radicals [37]. Additionally, γ-GT could also induce the generation of the endogenous ROS, which might accelerate the tumor proliferation and survival via aberrant CpG island methylation, DNA damage and genome instability [38]. From the other hand, tumor microenvironment could influence the expression of γ-GT. As a part of the tumor microenvironment, oxidative stress such as ROS could up-regulate γ-GT via the redox regulation of many genes [38], Moreover, inflammatory cytokines such as interferon-α/β and tumor necrosis factor α could also stimulate the expression of γ-GT [37, 39]. Hence, γ-GT could not only be a biomarker of the inflamed liver microenvironment, but also a biomarker of the prognosis, which indicated that γ-GT might guide the management of PA-TCAE.

However, there were several limitations in this study. First, selection bias and recalling bias were hard to avoid in a retrospective study, although PSM and multivariate cox model were conducted to decrease potential confounding factors. Second, interactions of γ-GT and PA-TACE might exist, but mechanism needed to be explored further. Third, the cut-off value in this study was the upper limit of the normal values, which might be different from each manufacture. The last but not the least, there are apparent differences between the West and East in the epidemiology, tumor characteristics, and management of HCC, which indicated that the conclusion needs further validation in the western series.

## Conclusion

Currently, we concluded that patients with more advanced HCC also have more elevated γ-GT, and these patients with elevated γ-GT would be benefited more from PA-TACE after radical resection. However, the conclusion needs further validation.

## Supplementary Information

**Additional file 1:.** Fig. S1 Comparison of overall survival (A) and disease-free survival (B) between the PA-TACE and non-TACE groups in the whole cohort

**Additional file 2: Fig. S2** Comparison of overall survival (A) and disease-free survival (B) according to the level of γ-GT in the whole cohort

**Additional file 3: Table S1** Clinicopathological characteristics before and after PSM in the group of **γ**-GT ≤ 54 U/L

**Additional file 4: Table S2** Clinicopathological characteristics before and after PSM in the group of **γ**-GT > 54 U/L

## Data Availability

All data included in this study are available upon request by contact with the corresponding author.

## Abbreviations

HCC: hepatocellular carcinoma
γ-GT: gamma-glutamyl transferase
PA-TACE: postoperative adjuvant transarterial chemoembolizations
OS: overall survival
DFS: disease-free survival
HR: Hazard ratio
CI: confidence interval
PSM: propensity scoring match
HBV: hepatitis B virus
TBil: total bilirubin
AFP: alpha-fetoprotein
MVI: microvascular invasion
BCLC: Barcelona Clinic Liver Cancer staging system
CNLC: China Liver cancer staging system
AJCC: the American Joint of Cancer Committee system
