# Supplementary figures and images for: Exploring the clinical value of preoperative serum gamma-glutamyl transferase levels in the management of patients with hepatocellular carcinoma receiving postoperative adjuvant transarterial chemoembolization
Source: BMC Cancer. 2021 Oct 18;21:1117. doi: 10.1186/s12885-021-08843-z (PMC8524816; doi:10.1186/s12885-021-08843-z)

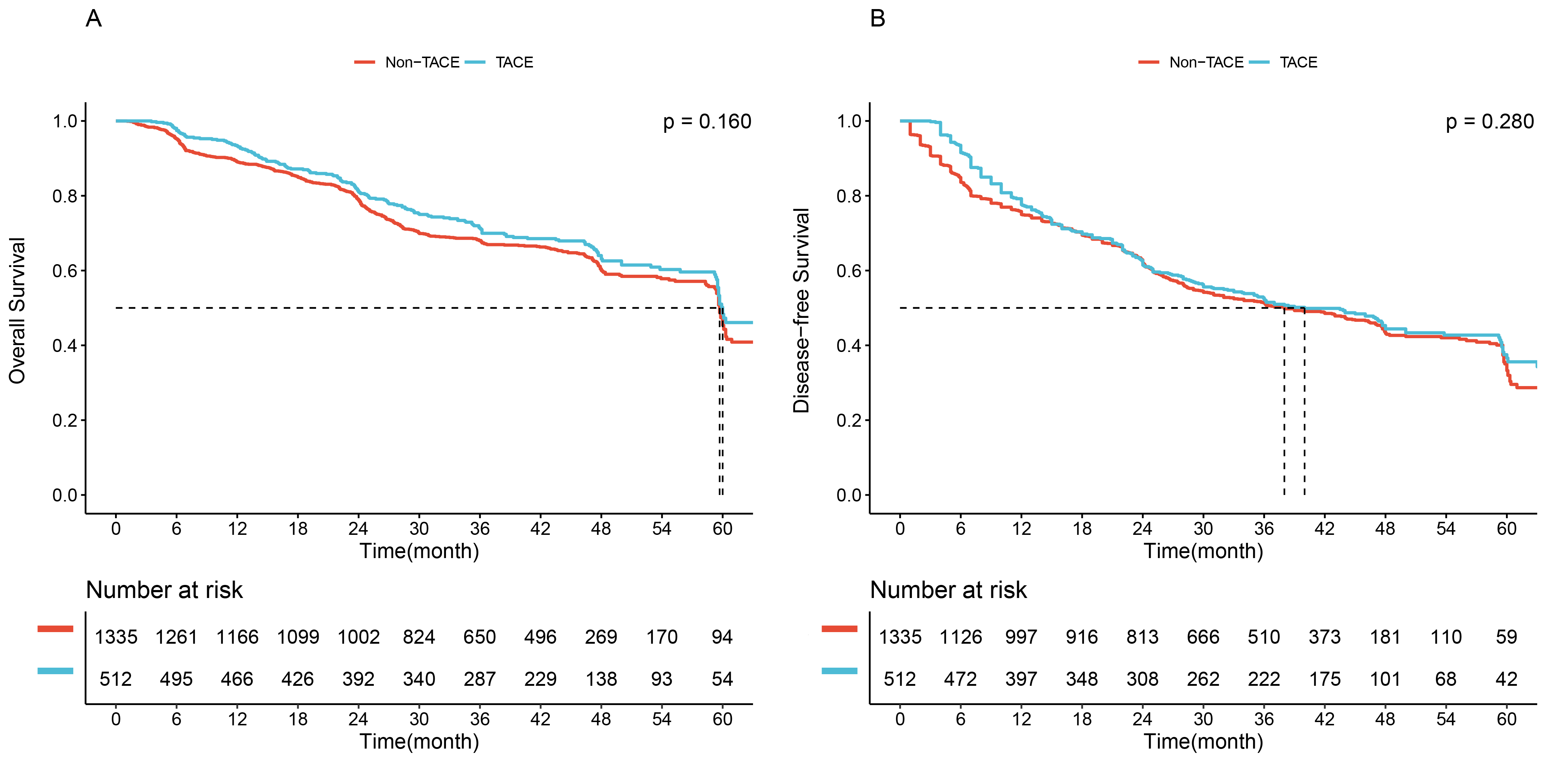

Supplement: Supplementary file 1 — Additional file 1:. Fig. S1 Comparison of overall survival (A) and disease-free survival (B) between the PA-TACE and non-TACE groups in the whole cohort [file 12885_2021_8843_MOESM1_ESM.tif]

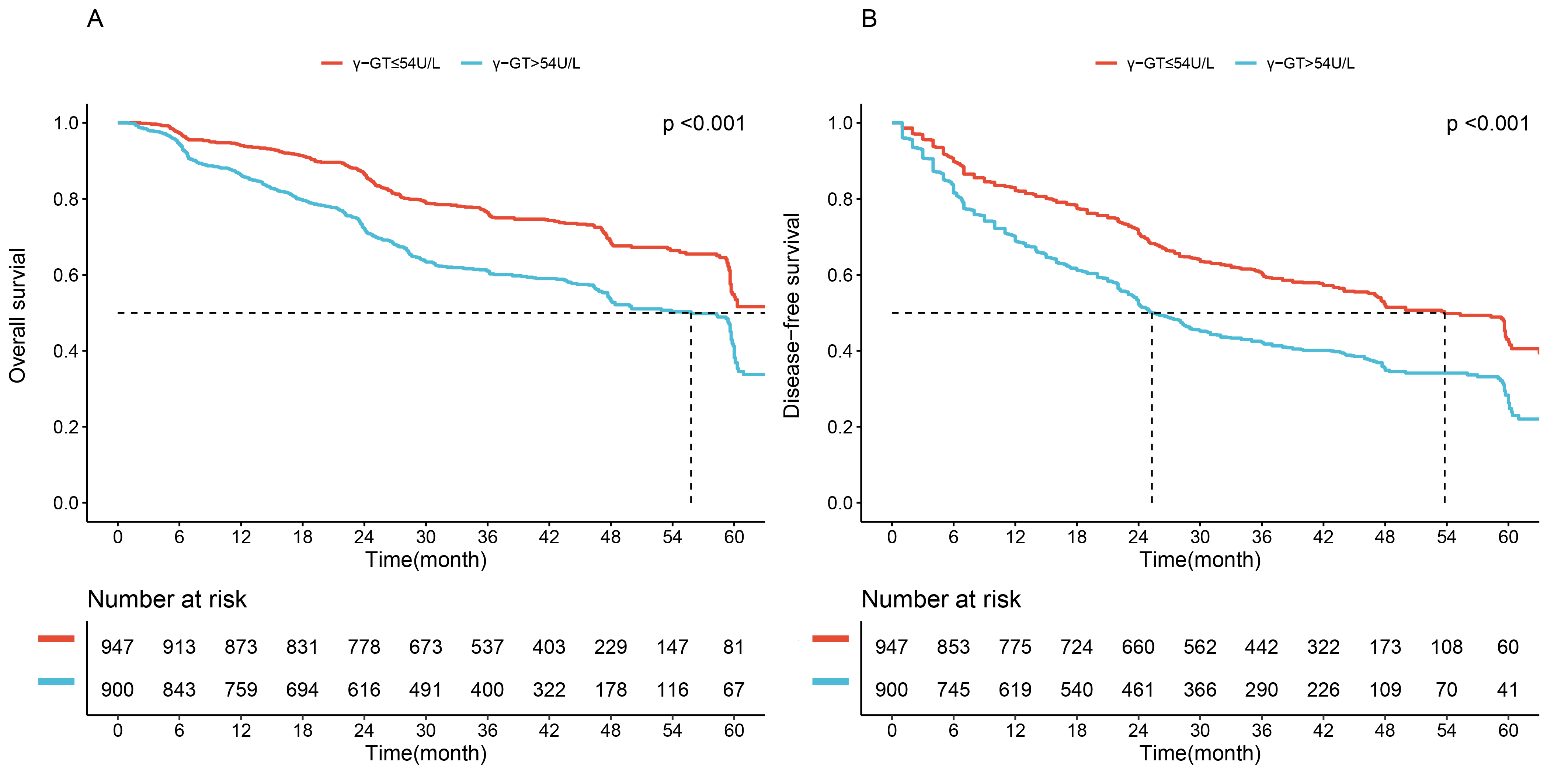

Supplement: Supplementary file 2 — Additional file 2: Fig. S2 Comparison of overall survival (A) and disease-free survival (B) according to the level of γ-GT in the whole cohort [file 12885_2021_8843_MOESM2_ESM.tif]
