# Supplementary material for: Exploring the clinical value of preoperative serum gamma-glutamyl transferase levels in the management of patients with hepatocellular carcinoma receiving postoperative adjuvant transarterial chemoembolization
Source: BMC Cancer. 2021 Oct 18;21:1117. doi: 10.1186/s12885-021-08843-z (PMC8524816; doi:10.1186/s12885-021-08843-z)
Supplement: Supplementary file 3 — Additional file 3: Table S1 Clinicopathological characteristics before and after PSM in the group of γ-GT ≤ 54 U/L [file 12885_2021_8843_MOESM3_ESM.docx]

| **Supplement Table 1.** Clinicopathological characteristics before and after PSM in the group of **γ**-GT≤54U/L | | | | | | | | |
| --- | --- | --- | --- | --- | --- | --- | --- | --- |
| **Characteristic** | | **Before PSM** | | |  | **After PSM** | | |
|  |  | **Non-TACE**  **（n=707）** | **PA-TACE**  **（n=240）** | ***P*-value** |  | **Non-TACE**  **（n=240）** | **PA-TACE**  **（n=240）** | ***P*-value** |
|  |  |  |  |  |  |  |  |  |
| **Age**(years) | Mean±SD | 52.4(10.6) | 52.0 (10.4) | 0.624 |  | 51.9 (10.5) | 52.0 (10.4) | 0.896 |
| **Sex** | Female | 139 (19.7%) | 43 (17.9%) | 0.619 |  | 44 (18.3%) | 43 (17.9%) | 1 |
|  | Male | 568 (80.3%) | 197 (82.1%) |  |  | 196 (81.7%) | 197 (82.1%) |  |
| **HBV infection** | No | 108 (15.3%) | 31 (12.9%) | 0.431 |  | 27 (11.2%) | 31 (12.9%) | 0.674 |
|  | Yes | 599 (84.7%) | 209 (87.1%) |  |  | 213 (88.8%) | 209 (87.1%) |  |
| **Cirrhosis** | No | 243 (34.4%) | 72 (30.0%) | 0.245 |  | 76 (31.7%) | 72 (30.0%) | 0.767 |
|  | Yes | 464 (65.6%) | 168 (68.3%) |  |  | 164 (68.3%) | 168 (68.3%) |  |
| **TBil**(μmol/L) | Mean±SD | 14.0 (5.49) | 14.8 (5.66) | 0.059 |  | 14.7 (5.62) | 14.8 (5.66) | 0.767 |
| **Child-pugh** | A | 692 (97.9%) | 233 (97.1%) | 0.647 |  | 236 (98.3%) | 233 (97.1%) | 0.542 |
|  | B | 15 (2.1%) | 7 (2.9%) |  |  | 4 (1.7%) | 7 (2.9%) |  |
| **AFP**(ng/ml) | ≤400 | 498 (70.4%) | 164 (68.3%) | 0.594 |  | 162 (67.5%) | 164 (68.3%) | 0.922 |
|  | >400 | 209 (29.6%) | 76 (31.7%) |  |  | 78 (32.5%) | 76 (31.7%) |  |
| **Transfusion** | No | 692 (97.9%) | 234 (97.5%) | 0.928 |  | 235 (97.9%) | 234 (97.5%) | 1 |
|  | Yes | 15 (2.1%) | 6 (2.5%) |  |  | 5 (2.1%) | 6 (2.5%) |  |
| **Tumor number** | Single | 614 (86.8%) | 185 (77.1%) | <0.001 |  | 186 (77.5%) | 185 (77.1%) | 1 |
|  | Multiple | 93 (13.2%) | 55 (22.9%) |  |  | 54 (22.5%) | 55 (22.9%) |  |
| **Tumor diameter**(cm) | Mean±SD | 4.49 (2.60) | 4.90 (2.72) | 0.043 |  | 4.93 (2.91) | 4.90 (2.72) | 0.908 |
| **ES grading** | I/II | 75 (10.6%) | 24 (10.0%) | 0.885 |  | 26 (10.8%) | 24 (10.0%) | 0.881 |
|  | III/IV | 632 (89.4%) | 216 (90.0%) |  |  | 214 (89.2%) | 216 (90.0%) |  |
| **Capsule** | Present | 572 (80.9%) | 198 (82.5%) | 0.651 |  | 186 (77.5%) | 198 (82.5%) | 0.209 |
|  | Absent | 135 (19.1%) | 42 (17.5%) |  |  | 54 (22.5%) | 42 (17.5%) |  |
| **Satellite** | No | 441 (62.4%) | 131 (54.6%) | 0.040 |  | 138 (57.5%) | 131 (54.6%) | 0.581 |
|  | Yes | 266 (37.6%) | 109 (45.4%) |  |  | 102 (42.5%) | 109 (45.4%) |  |
| **BCLC stage** | 0 | 62 (8.8%) | 17 (7.1%) | 0.015 |  | 24 (10.0%) | 17 (7.1%) | 0.500 |
|  | A | 573 (81.0%) | 182 (75.8%) |  |  | 174 (72.5%) | 182 (75.8%) |  |
|  | B | 72 (10.2%) | 41 (17.1%) |  |  | 42 (17.5%) | 41 (17.1%) |  |
| **AJCC** | Ia | 62 (8.8%) | 17 (7.1%) | 0.032 |  | 24 (10.0%) | 17 (7.1%) | 0.369 |
|  | Ib | 386 (54.6%) | 109 (45.4%) |  |  | 111 (46.2%) | 109 (45.4%) |  |
|  | II | 226 (32.0%) | 96 (40.0%) |  |  | 84 (35.0%) | 96 (40.0%) |  |
|  | IIIa | 33 (4.7%) | 18 (7.5%) |  |  | 21 (8.8%) | 18 (7.5%) |  |
| **CNLC stage** | Ia | 433 (61.2%) | 116 (48.3%) | <0.001 |  | 112 (46.7%) | 116 (48.3%) | 0.972 |
|  | Ib | 202 (28.6%) | 83 (34.6%) |  |  | 86 (35.8%) | 83 (34.6%) |  |
|  | IIa | 60 (8.5%) | 30 (12.5%) |  |  | 32 (13.3%) | 30 (12.5%) |  |
|  | IIb | 12 (1.7%) | 11 (4.6%) |  |  | 10 (4.2%) | 11 (4.6%) |  |
| Note: HBV, hepatitis B virus, TBil, total bilirubin; AFP, alpha-fetoprotein; γ-GT, gamma-glutamyl transferase; ES, Edmondson-Steiner; MVI, microvascular invasion; PA-TACE, postoperative adjuvant transarterial chemoembolization; AJCC, according to the 8th American joint committee on cancer staging; CNLC, CNLC staging was defined according the Chinese guideline for HCC. | | | | | | | | |
