# Supplementary material for: Exploring the clinical value of preoperative serum gamma-glutamyl transferase levels in the management of patients with hepatocellular carcinoma receiving postoperative adjuvant transarterial chemoembolization
Source: BMC Cancer. 2021 Oct 18;21:1117. doi: 10.1186/s12885-021-08843-z (PMC8524816; doi:10.1186/s12885-021-08843-z)
Supplement: Supplementary file 4 — Additional file 4: Table S2 Clinicopathological characteristics before and after PSM in the group of γ-GT > 54 U/L [file 12885_2021_8843_MOESM4_ESM.docx]

| **Supplement Table 2.** Clinicopathological characteristics before and after PSM in the group of **γ**-GT>54U/L | | | | | | | | |
| --- | --- | --- | --- | --- | --- | --- | --- | --- |
| **Characteristic** | | **Before PSM** | | |  | **After PSM** | | |
|  |  | **Non-TACE**  **（n=628）** | **PA-TACE**  **（n=272）** | ***P*-value** |  | **Non-TACE**  **（n=272）** | **PA-TACE**  **（n=272）** | ***P*-value** |
|  |  |  |  |  |  |  |  |  |
| **Age**(years) | Mean±SD | 51.6 (10.5) | 53.3 (10.8) | 0.030 |  | 53.1(10.2) | 53.3 (10.8) | 0.825 |
| **Sex** | Female | 65 (10.4%) | 25 (9.2%) | 0.681 |  | 33 (12.1%) | 25 (9.2%) | 0.331 |
|  | Male | 563 (89.6%) | 247 (90.8%) |  |  | 239(87.9%) | 247 (90.8%) |  |
| **HBV infection** | No | 82 (13.1%) | 21 (7.7%) | 0.028 |  | 20 (7.4%) | 21 (7.7%) | 1 |
|  | Yes | 546 (86.9%) | 251 (92.3%) |  |  | 252(92.6%) | 251 (92.3%) |  |
| **Cirrhosis** | No | 216 (34.4%) | 79 (29.0%) | 0.135 |  | 86 (31.6%) | 79 (29.0%) | 0.576 |
|  | Yes | 412 (65.6%) | 193 (71.0%) |  |  | 186(68.4%) | 193 (71.0%) |  |
| **TBil**(μmol/L) | Mean±SD | 15.0 (6.80) | 15.1 (6.76) | 0.829 |  | 15.3 (7.03) | 15.1 (6.76) | 0.837 |
| **Child-pugh** | A | 576 (91.7%) | 250 (91.9%) | 1 |  | 247(90.8%) | 250 (91.9%) | 0.760 |
|  | B | 52 (8.3%) | 22 (8.1%) |  |  | 25 (9.2%) | 22 (8.1%) |  |
| **AFP**(ng/ml) | ≤400 | 426 (67.8%) | 167 (61.4%) | 0.073 |  | 162(59.6%) | 167 (61.4%) | 0.726 |
|  | >400 | 202 (32.2%) | 105 (38.6%) |  |  | 110 (40.4%) | 105 (38.6%) |  |
| **Transfusion** | No | 569 (90.6%) | 244 (89.7%) | 0.767 |  | 237(87.1%) | 244 (89.7%) | 0.421 |
|  | Yes | 59 (9.4%) | 28 (10.3%) |  |  | 35 (12.9%) | 28 (10.3%) |  |
| **Tumor number** | Single | 509 (81.1%) | 191 (70.2%) | <0.001 |  | 201 (73.9%) | 191 (70.2%) | 0.390 |
|  | Multiple | 119 (18.9%) | 81 (29.8%) |  |  | 71 (26.1%) | 81 (29.8%) |  |
| **Tumor diameter**(cm) | Mean±SD | 6.99 (4.38) | 7.58 (4.20) | 0.056 |  | 7.93(4.75) | 7.58 (4.20) | 0.368 |
| **ES grading** | I/II | 51 (8.1%) | 22 (8.1%) | 1 |  | 12 (4.4%) | 22 (8.1%) | 0.111 |
|  | III/IV | 577 (91.9%) | 250 (91.9%) |  |  | 260 (95.6%) | 250 (91.9%) |  |
| **Capsule** | Present | 493 (78.5%) | 224 (82.4%) | 0.220 |  | 220 (80.9%) | 224 (82.4%) | 0.740 |
|  | Absent | 135 (21.5%) | 48 (17.6%) |  |  | 52 (19.1%) | 48 (17.6%) |  |
| **Satellite** | No | 361 (57.5%) | 138 (50.7%) | 0.072 |  | 147 (54.0%) | 138 (50.7%) | 0.492 |
|  | Yes | 267 (42.5%) | 134 (49.3%) |  |  | 125 (46.0%) | 134 (49.3%) |  |
| **BCLC stage** | 0 | 26 (4.1%) | 2 (0.7%) | <0.001 |  | 7 (2.6%) | 2 (0.7%) | 0.240 |
|  | A | 492 (78.3%) | 199 (73.2%) |  |  | 197 (72.4%) | 199 (73.2%) |  |
|  | B | 110 (17.5%) | 71 (26.1%) |  |  | 68 (25.0%) | 71 (26.1%) |  |
| **AJCC** | Ia | 26 (4.1%) | 2 (0.7%) | 0.039 |  | 7 (2.6%) | 2 (0.7%) | 0.257 |
|  | Ib | 313 (49.8%) | 130 (47.8%) |  |  | 132 (48.5%) | 130 (47.8%) |  |
|  | II | 213 (33.9%) | 88 (32.4%) |  |  | 87 (32.0%) | 88 (32.4%) |  |
|  | IIIa | 76 (12.1%) | 52 (19.1%) |  |  | 46 (16.9%) | 52 (19.1%) |  |
| **CNLC stage** | Ia | 231 (36.8%) | 66 (24.3%) | <0.001 |  | 69 (25.4%) | 66 (24.3%) | 0.984 |
|  | Ib | 287 (45.7%) | 135 (49.6%) |  |  | 135 (49.6%) | 135 (49.6%) |  |
|  | IIa | 68 (10.8%) | 45 (16.5%) |  |  | 44 (16.2%) | 45 (16.5%) |  |
|  | IIb | 42 (6.7%) | 26 (9.6%) |  |  | 24 (8.8%) | 26 (9.6%) |  |
| Note: HBV, hepatitis B virus, TBil, total bilirubin; AFP, alpha-fetoprotein; γ-GT, gamma-glutamyl transferase; ES, Edmondson-Steiner; MVI, microvascular invasion; PA-TACE, postoperative adjuvant transarterial chemoembolization; AJCC, according to the 8th American joint committee on cancer staging; CNLC, CNLC staging was defined according the Chinese guideline for HCC. | | | | | | | | |
